# Supplementary material for: Optimization of negative stage bias potential for faster imaging in large-scale electron microscopy
Source: J Struct Biol X. 2021 Feb 9;5:100046. doi: 10.1016/j.yjsbx.2021.100046 (PMC7973379; doi:10.1016/j.yjsbx.2021.100046)
Supplement: Supplementary data 2 [file mmc2.docx]

| **Initial BSE energy (eV)** | **Imaging mode** | **Stage bias (V)** | **Working distance (mm)** | **BSE bundle size** |
| --- | --- | --- | --- | --- |
| 500 1000 1500 2000 3000 | HR UHR | 0 100 500 1000 1500 2000 3000 | 3 4 5 6 | 100 particles |

Table 1. **Parameter space available for tracing BSE trajectories**. To estimate BSE yield, code for calculating the radial distance of individual backscattered electrons at a given detector plane is available in Appendix A.
